# The characteristics of intestinal microbiota in patients with chronic schistosomiasis *japonica*-induced liver fibrosis by 16S rRNA gene sequence

**DOI:** 10.3389/fmicb.2023.1276404

**Published:** 2023-10-03

**Authors:** Chen Guo, Pengpeng Zhang, Junhui Li, Chen Zhou, Zhen Yang, Yu Zhang, Yulin Luo, Jie Zhou, Yu Cai, Yingzi Ming

**Affiliations:** ^1^Organ Transplantation Center, The Third Xiangya Hospital of Central South University, Changsha, Hunan, China; ^2^Engineering and Technology Research Center for Transplantation Medicine of the National Ministry of Health, The Third Xiangya Hospital of Central South University, Changsha, Hunan, China; ^3^Hunan Institute of Schistosomiasis Control, Yueyang, Hunan, China

**Keywords:** chronic schistosomiasis *japonica*, liver fibrosis, intestinal microbiota, 16S rRNA gene sequence, *Prevotella* 7, *Alloprevotella*, *Holdemanella*

## Abstract

**Background:**

The intestinal microbiota is known to play a role in the development of liver disease, there is a limited understanding of the intestinal microbiota associated with chronic schistosomiasis *japonica*. This study sought to explore the characteristics of the intestinal microbiota in patients with chronic schistosomiasis *japonica* and identify potential biomarkers that could aid diagnosis.

**Methods:**

A total of 40 residents of Qingshan Island in Yueyang (Hunan, China) were enrolled in this cross-sectional study. These individuals were divided into two groups for analysis of the intestinal microbiota: patients with chronic schistosomiasis *japonica*-induced liver fibrosis group (CSJ group, *n* = 10) and a healthy control group (HC group, *n* = 30). Feces were collected from each participant and analyzed by 16S rRNA gene sequencing, which included species composition analysis at the phylum and family levels, α and β diversity analysis, LEfSe, Kyoto Encyclopedia of Genes and Genome (KEGG) and Clusters of Orthologous Groups of proteins (COG) analysis.

**Results:**

Our results indicated that *Schistosoma japonicum* infection changed the composition and abundance of intestinal microbiota at the phylum and family levels. Compared with the HC group, the α and β diversity results showed that CSJ group had low diversity of species of the intestinal microbiome. LEfSe and relative abundance analysis found that the *Prevotella* 7, *Alloprevotella*, and *Holdemanella* genera were significantly higher in the CSJ group than in the HC group. Meanwhile, the ROC analysis showed that the area under the curve (AUC) of *Prevotella* 7, *Alloprevotella*, and *Holdemanella* genera was 0.779, 0.769, and 0.840, respectively. KEGG and COG analysis showed that the Replication and Repair, and Defense Mechanism pathways correlated strongly with chronic schistosomiasis *japonica* infection.

**Conclusion:**

The current study was the first to explore differences in the intestinal microbiota of patients with chronic schistosomiasis *japonica*-induced liver fibrosis and healthy people from Qingshan Island, which indicated that *Prevotella* 7, *Alloprevotella*, and *Holdemanella* genera could have a potential value in non-invasive diagnosis of chronic schistosomiasis *japonica*-induced fibrosis.

## 1. Introduction

Schistosomiasis is a tropical disease worldwide. An estimated 779 million people are threatened by Schistosoma infection and over 250 million people were infected with *Schistosoma* spp., resulting in about 1.9 million disability-adjusted life years (DALYs) (Steinmann et al., 2006; Utzinger et al., 2009; GBD 2016 DALYs and HALE Collaborators, 2017). *Schistosoma japonicum* is endemic in China, especially in the Dongting Lake District (Li et al., 2020; Wang et al., 2021). Qingshan Island, previously called Qingtan Town, is an isolated island in the South Dongting Lake District that has an *S. japonicum* infection rate of 15.8% in 1998, which is detected by Kato–Katz method and miracidia hatching test (Yan et al., 2003), providing a large patient cohort for the study of this disease.

Chronic schistosomiasis *japonica* is primarily caused by the host’s reaction to eggs produced by the female Schistosoma, not by the worms themselves. Female *S. japonicum* can produce up to 3,000 eggs per day, some of which are deposited in the portal area of the liver, where they elicit a Th2-mediated immune response by the host; over time, the chronic local immune response leads to the formation of granulomas, irreversible tissue fibrosis generation, and cirrhosis, which can threaten the life of the patient (Colley et al., 2014; McManus et al., 2018, 2020; Llanwarne and Helmby, 2021). Schistosomiasis *japonica* includes acute, chronic, and advanced stages. Patients with chronic infection may be slightly symptomatic or symptomless. If not treated in time, chronic infection may develop to advanced schistosomiasis, which has a high mortality (Chen, 2014). Thus, inhibiting the transition from chronic to advanced schistosomiasis *japonica* is essential to effectively reduce disease-associated mortality. However, the lack of a reliable diagnostic method of patients with chronic infection has delayed the onset of treatment and resulted in poorer outcomes (Mao, 1990; LoVerde, 2019). Currently, both parasitology- and serology-based methods are used to diagnose *S. japonicum* infection. Those methods had several disadvantages, including a high rate of cross-reactivity with other parasitic diseases, false-positive results, and difficulty in distinguishing current from previous infections (Bergquist et al., 2017; Chen et al., 2021). Thus, there is a clear need to explore and develop a non-invasive and highly sensitive diagnostic method to identify chronic schistosomiasis *japonica*-induced liver fibrosis.

The intestinal microbiota plays a major role in the regulation of the host immune functions and maintaining immune homeostasis (Belkaid and Harrison, 2017; Pacaud et al., 2021). The intestinal microbiome dysbiosis has been associated with several immune diseases, including type II diabetes, inflammatory bowel disease, and multiple sclerosis (Lavelle and Sokol, 2020; Ghezzi et al., 2021; Yang et al., 2021). Liver diseases are also closely related to an imbalance in the intestinal microbiome since the liver secretes bile and other substances that interact directly with intestinal microbiota (Jones and Neish, 2021). Recent studies have shown that the intestinal microbiota becomes altered during acute and advanced schistosomiasis, indicating that the microbiome could serve as a marker for this disease (Gui et al., 2021; Jiang et al., 2021). Moreover, an intervention using *Bacillus subtilis* was shown to reduce liver fibrosis and other related symptoms in *S. japonicum*-infected mice (Lin et al., 2021). These findings suggest that intestinal microbiota may aid the diagnosis and treatment of schistosomiasis. However, knowledge about the characteristics of the intestinal microbiota in patients with chronic schistosomiasis *japonica*-induced liver fibrosis remains limited. The current study conducted an intestinal microbiota analysis of patients with chronic schistosomiasis *japonica*-induced liver fibrosis and healthy individuals from Qingshan Island. This study explored the characteristics of the intestinal microbiota associated with chronic schistosomiasis *japonica*-induced liver fibrosis and provided data that could be used to inform more effective diagnostic methods and treatments for this disease.

## 2. Materials and methods

### 2.1. Participants and samples collection

This study was conducted in May 2022. A total of 56 inhabitants living on Qingshan Island were engaged into the cross-sectional study, of whom 40 were finally enrolled for intestinal microbiota analysis and divided into two groups: patients with chronic schistosomiasis *japonica*-induced liver fibrosis group (CSJ group, *n* = 10) and a healthy control group (HC group, *n* = 30). CSJ participants were eligible for inclusion in the study if they met the following criteria: (a) with a clear history of *S. japonicum* infection; (b) history of treatment for *S. japonicum*; (c) have no prior history anti-helminthic or antibiotic treatment within 1 month (Schneeberger et al., 2018; Ramirez et al., 2020); (d) with liver fibrosis by ultrasound diagnosis, without ascites, portal hypertension and splenomegaly, etc., which referred to the previous studies (Abdel Wahab et al., 1992; Richter et al., 2003; Wu et al., 2015). Participants were excluded if they presented with cognitive or mental disabilities, gastrointestinal diseases, and/or other liver diseases, such as hepatitis, fatty liver and hepatocellular carcinoma. HC individuals were referred to reside on Qingshan Island, have no prior history of *S. japonicum* and test negative for *S. japonicum*. Fresh feces and blood were collected from all participants for analysis. Blood routine examination, including hemoglobin (HGB), neutrophils (NEU), eosinophils (EOS), lymphocyte (MON) and platelet (PLT), were tested by an automated hematologic analyzer (Sysmex XN-9000); Blood biochemical examination, including total triglycerides (TG), total cholesterol (TC), alanine aminotransferase (ALT), aspartate aminotransferase (AST), alkaline phosphatase (ALP), albumin (ALB), globulin (GLOB), and total protein (TP) were tested by an automatic biochemical analyzer (Hitachi 7600).

### 2.2. Extraction of genome DNA

Total genomic DNA from fecal samples were extracted using OMEGA Soil DNA Kit (M5635-02, Omega Bio-Tek). DNA concentration and purity were monitored on 1% agarose gels. According to the concentration, DNA was diluted to 1 ng/μl using sterile water.

### 2.3. Amplicon generation

The V3–4 hypervariable region of the 16S rRNA genes was amplified using the specific primers with the barcode: 341F (5′-CCTAYGGGRBGCASCAG-3′) and 806R (5′-GGACTACNNGGGTATCTAAT-3′). All PCR reactions were carried out in 30 μl reactions with 15 μl of the Phusion^®^ High-Fidelity PCR Master Mix (New England Biolabs), 0.2 μM of the forward and reverse primers, and about 10 ng template DNA. Thermal cycling included an initial denaturation of 98°C for 1 min, followed by 30 cycles of denaturation at 98°C for 10 s, annealing at 50°C for 30 s, elongation at 72°C for 60 s, and finally heat preservation for 5 min at 72°C.

### 2.4. PCR product quantification and qualification

The same volume of 1× loading buffer (containing SYB green) was mixed with the PCR products and subjected to electrophoresis on a 2% agarose gel. Samples with a bright strip between 400 and 450 bp were chosen for further experiments. PCR products were mixed in equidensity ratios. Then, mixture PCR products was purified with Qiagen Gel Extraction Kit (Qiagen).

### 2.5. Library preparation and sequencing

Sequencing libraries were generated using TruSeq^®^ DNA PCR-Free Sample Preparation Kit (Illumina) following manufacturer’s recommendations and index codes were added. The library quality was assessed on the Qubit^®^ 2.0 Fluorometer (Thermo Scientific) and Agilent Bioanalyzer 2100 system. At last, the library was sequenced on an Illumina NovaSeq6000 platform and 250 bp paired-end reads were generated. Quality control on the raw reads was performed using Quantitative Insights into Microbial Ecology (QIIME 1.8.0) as previously described (Caporaso et al., 2010).

### 2.6. Sequencing data analysis

Paired-end reads from the original DNA fragments were merged using FLASH (Magoè and Salzberg, 2011), a very fast and accurate analysis tool designed to merge paired-end reads. When at least some of the reads overlap the read was generated from the opposite end of the same DNA fragment. Paired-end reads were assigned to each sample according to the unique barcodes. Sequence analysis was performed using the UPARSE software package with the UPARSE-Operational Taxonomic Units (OTU) and UPARSE-OTUref algorithms (Edgar, 2010, 2013). Sequences with ≥97% similarity were assigned to the same OTUs. A representative sequence was chosen for each OTU and the RDP classifier was used to annotate taxonomic information for each representative sequence (Silva 132 for 16S, UNITE for ITS). α (within sample) and β (among sample) diversities were estimated using MOTHUR software (v1.31.2) (Schloss et al., 2009) and the QIIME pipeline (v1.8.0), respectively. The Observed Species, Good’s coverage, Chao1, ACE, Shannon, and Simpson indexes were used to evaluate α-diversity. Shannon curves were generated based on these indexes. β-diversity analysis was calculated using the weighted UniFrac (Lozupone et al., 2011) and visualized by principal coordinates analysis (PCoA) downscaling. To measure differences in individual taxonomic abundance, linear discriminant analysis (LDA) effect size (LEfSe) was used to count species with significant differences in community structure between the samples. A LDA score >2 was considered a significant difference and used to screen for characteristic biomarkers of the intestinal microbiota between groups (Segata et al., 2011). Microbial functions were predicted by PICRUSt (phylogenetic investigation of communities by reconstruction of unobserved states) (Langille et al., 2013) in the Kyoto Encyclopedia of Genes and Genomes (KEGG) and Clusters of Orthologous Groups of proteins (COG) databases based on 16S rRNA gene sequence data. KEGG and COG analysis between two groups were conducted using LEfSe (Segata et al., 2011).

### 2.7. Statistical analysis

Statistical analyses were performed using SPSS software (v 26.0; SPSS, IL, USA). Continuous variables were expressed as the mean ± standard deviation (SD) or median [interquartile range (IQR)]. The Student’s *t*-test or Wilcoxon Rank-Sum and the χ^2^ tests were used to evaluate differences between two groups for continuous and categorical variables, respectively. Receiver operation characteristic (ROC) analysis to predict the diagnostic efficiency of the different intestinal microbiota. The false discovery rate (FDR) was used to correct the calculated *Q*-values. A *P*-value < 0.05 was considered statistically significant.

## 3. Results

### 3.1. Participant characteristics

**FIGURE 1:**
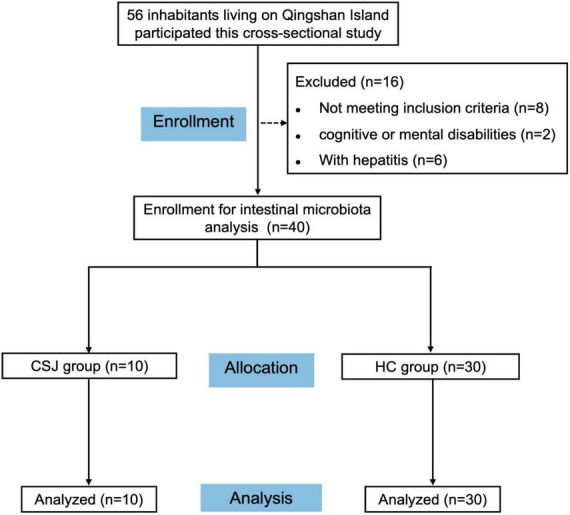
Flow diagram depicting participant recruitment and retention. A total of 56 inhabitants were engaged into the cross-sectional study, of whom 16 were excluded, and 40 were finally enrolled for intestinal microbiota analysis and divided into chronic schistosomiasis *japonica*-induced liver fibrosis group (CSJ group) and a healthy control group (HC group).

A participant flow chart was shown in Figure 1. A total of 56 inhabitants of Qingshan Island participated in the cross-sectional study, of whom 40 were finally enrolled in the intestinal microbiota analysis based on the inclusion and exclusion criteria. These participants were divided into a CSJ group (*n* = 10) and a HC group (*n* = 30). The general characteristics of all participants were shown in Table 1, no significant differences were observed between the two groups.

**TABLE 1:** Demographic and clinical characteristics of participants.

Variables	CSJ group (*n* = 10)	HC group (*n* = 30)	Statistical analysis
Gender (M/F)	3/7	11/19	χ2 = 0.15, df = 1, *P* = 0.70
Age (years)	59.8 ± 6.4	61.1 ± 9.6	*t*_(38)_ = 0.39, *P* = 0.21
BMI (kg/m^2^)	22.7 ± 2.4	24.1 ± 4.1	*Z* = −1.00, *P* = 0.32
Active smoker (%)	40	20	χ2 = 1.60, df = 1, *P* = 0.21
Active drinker (%)	30	20	χ2 = 0.43, df = 1, *P* = 0.51
TG (mmol/L)	1.8 ± 1.1	2.5 ± 1.1	*t*_(38)_ = 1.67, *P* = 0.96
TC (mmol/L)	5.3 ± 1.0	5.9 ± 0.8	*t*_(38)_ = 2.26, *P* = 0.28
ALT (U/L)	18.2 ± 4.1	18.4 ± 5.8	*Z* = −0.09, *P* = 0.92
AST (U/L)	34.8 ± 5.8	33.9 ± 9.9	*Z* = −1.22, *P* = 0.22
ALP (U/L)	83.6 ± 23.9	88.3 ± 18.3	*t*_(38)_ = 0.65, *P* = 0.22
ALB (g/L)	41.8 ± 2.7	42.4 ± 2.1	*t*_(38)_ = 0.63, *P* = 0.51
GLOB (g/L)	27.0 ± 3.9	27.4 ± 4.2	*t*_(38)_ = 0.24, *P* = 0.90
TP (g/L)	68.9 ± 4.7	69.8 ± 4.1	*t*_(38)_ = 0.56, *P* = 0.46
HGB (%)	136.8 ± 11.5	138.7 ± 11.4	*t*_(38)_ = 0.46, *P* = 0.85
NEU (%)	58.8 ± 8.8	57.8 ± 7.6	*t*_(38)_ = −0.35, *P* = 0.61
EOS (%)	2.1 ± 1.5	2.2 ± 1.2	*t*_(38)_ = 0.29, *P* = 0.45
LYM (%)	31.8 ± 8.9	33.5 ± 8.0	*t*_(38)_ = 0.58, *P* = 0.84
MOM (%)	6.9 ± 0.7	6.8 ± 0.9	*t*_(38)_ = −0.42, *P* = 0.11
PLT	159.9 ± 55.8	181.5 ± 46.9	*t*_(38)_ = 1.20, *P* = 0.51

BMI, body mass index; TG, total triglycerides; TC, total cholesterol; ALT, alanine aminotransferase; AST, aspartate aminotransferase; ALP, alkaline phosphatase; ALB, albumin; GLOB, globulin; TP, total protein; HGB, hemoglobin; NEU, neutrophils; EOS, eosinophils; LYM, lymphocyte; MON, monocyte; PLT, platelet count; CSJ, chronic schistosomiasis *japonica*; HC, healthy control.

### 3.2. Species composition and abundance of the intestinal microbiota in the two groups

**FIGURE 2:**
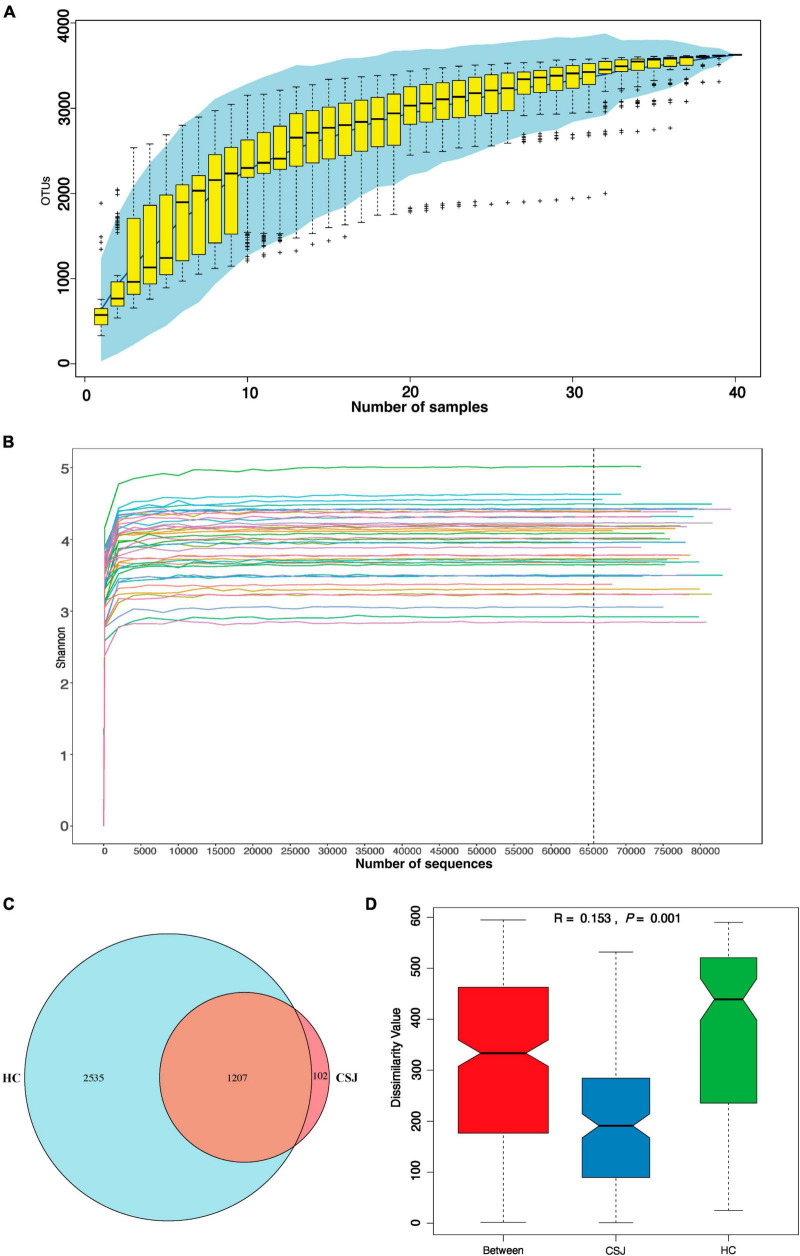
Quality evaluation of 16S rRNA gene sequence data. **(A)** Accumulation curve was given by cumulative data of operational taxonomic units (OTUs) per sample. **(B)** Shannon curve was constructed according to the microbial diversity index of the sequencing quantity of each sample at different sequencing depths. **(C)** Venn diagram represented the OTUs detected each sample between two groups and indicated that 1,207 of these OTUs were found in both groups. **(D)** Analysis of similarities (ANOSIM) was based on Bray–Curtis algorithm, *R* > 0 was considered a significant difference between groups rather than within groups, *P* < 0.05 was considered to be statistically significant.

In our study, 250 bp paired-end reads were generated by Illumina NovaSeq6000 platform. There were 3,060,101 sequences in total after demultiplexing and filtering for read quality, with a median of 77,322 sequences per experimental sample. The species accumulation curve of the samples reached a plateau, indicating that the study sample size was sufficient (Figure 2A). The Shannon curve indicated that the sequencing depth detected the most microbial information for each fecal sample (Figure 2B). To remove potential signal from contaminants, we removed from analysis taxa with >10% relative abundance in the blank swab samples and OTUs with <2 total occurrences among the samples. All reads clustered into 5,051 OTUs. Among them, 1,309 and 3,742 OTUs were detected in the CSJ and HC groups, respectively, and a Venn diagram indicated that 1,207 of these OTUs were found in both groups (Figure 2C). ANOSIM analysis was also performed and the between-group difference was greater than the within-group difference (*R* = 0.153, *P* = 0.001), indicating that the study groups were valid (Figure 2D).

**FIGURE 3:**
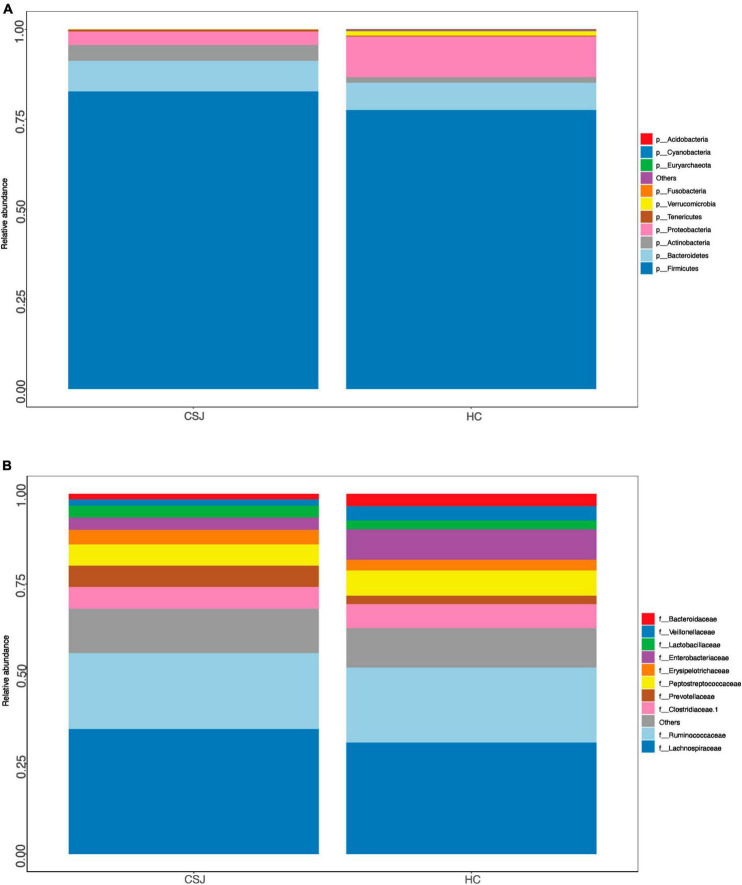
Species distribution of intestinal microbiota between CSJ group and HC group on phylum level **(A)** and family level **(B)**.

Relative abundance of the intestinal microbiota at the phylum and family levels was also assessed. At the phylum level, the dominant microbiota of the two groups were similar, indicating that the disease status did not change the “Enterotype” of the patients. *Firmicutes*, *Bacteroidetes*, and *Proteobacteria* were the three most common phyla found in both groups, together accounting for 94.9 and 96.4% of the abundance in the CSJ group and HC groups, respectively. The abundance of *Firmicutes* and *Bacteroidetes* was higher in the CSJ group than in the HC group, while the abundance of *Proteobacteria* was significantly higher in the HC group than in the CSJ group (Figure 3A). At the family level, Lachnospiraceae, Prevotellaceae, and Erysipelotrichaceae were increased in CSJ group, while Enterobacteriaceae and Veillonellaceae were reduced (Figure 3B).

### 3.3. The diversity analysis of the intestinal microbiota in the two groups

#### 3.3.1. α-Diversity analysis

**FIGURE 4:**
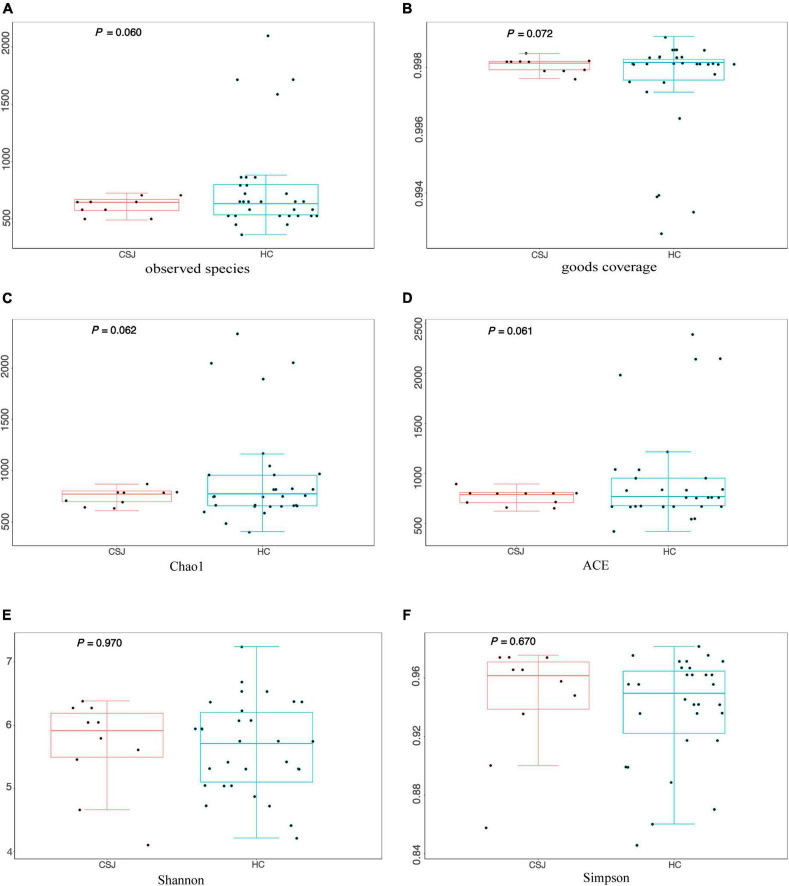
The α-diversity analysis of intestinal microbiota was evaluated by six metrics: **(A)** Observed Species index (*Z* = –1.55, *P* = 0.060); **(B)** Good’s coverage index (*Z* = –1.47, *P* = 0.072); **(C)** Chao1 index (*Z* = –1.54, *P* = 0.062); **(D)** ACE index (*Z* = –1.54, *P* = 0.061); **(E)** Shannon index (*t*_(38)_ = 0.69, *P* = 0.970); **(F)** Simpson index (*Z* = –1.50, *P* = 0.670).

α-Diversity represented the diversity of species within a given community. The following indexes were used to evaluate α-diversity: Observed Species (*Z* = −1.55, *P* = 0.060), Good’s coverage (*Z* = −1.47, *P* = 0.072), Chao1 (*Z* = −1.54, *P* = 0.062), ACE (*Z* = −1.54, *P* = 0.061), Shannon (*t*_(38)_ = 0.69, *P* = 0.970), and Simpson (*Z* = −1.50, *P* = 0.670). Differences were detected between the two groups in four indexes except Shannon and Simpson indexes, indicating that CSJ group had lower diversity of species (Figure 4).

#### 3.3.2. β-Diversity analysis

**FIGURE 5:**
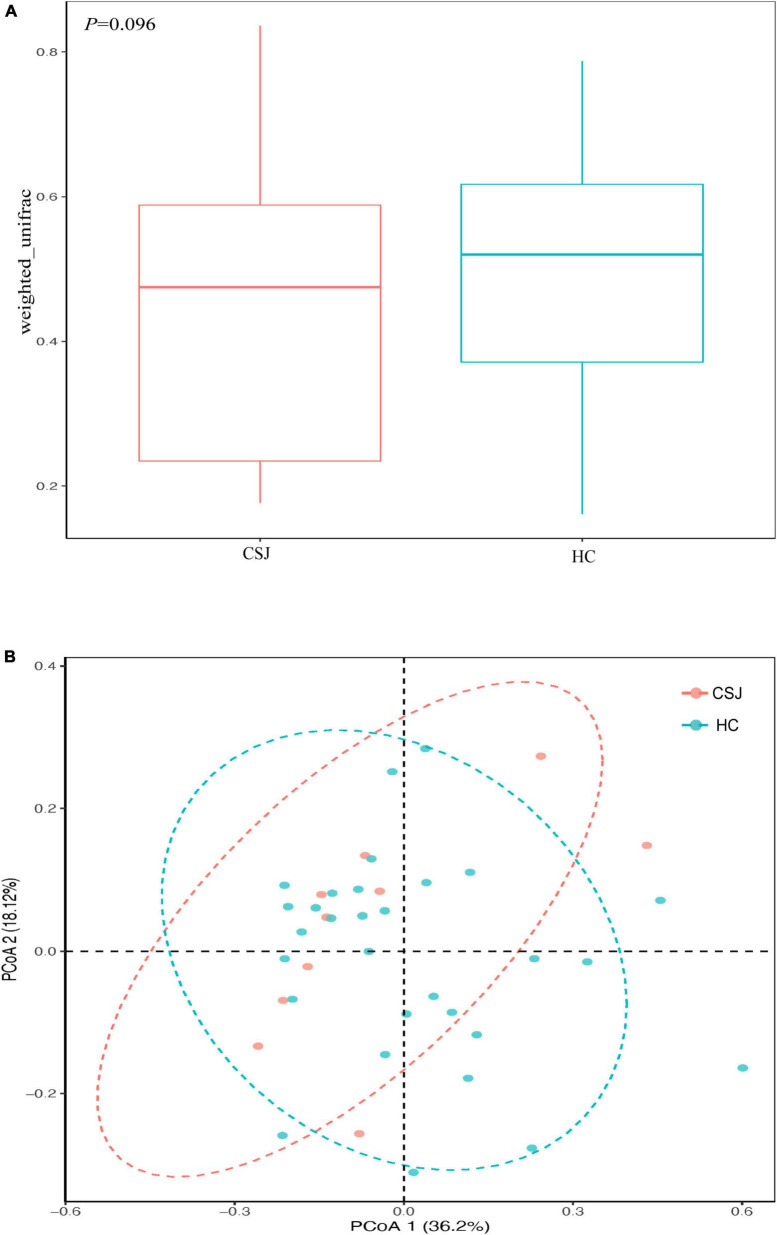
The β-diversity analysis of intestinal microbiota was analyzed based on weighted UniFrac (*Z* = –1.30, *P* = 0.096) **(A)** and PCoA **(B)** between the CSJ group and HC group.

β-Diversity was used to assess the diversity of intestinal microbiota communities based on weighted UniFrac distances and PCoA analysis. It had trend to reach the significant difference in β-diversity between two groups (*Z* = −1.30, *P* = 0.096) (Figure 5A). Meanwhile, the PCoA results indicated that samples from each group had a species-specific heterogeneous distribution, suggesting that the diversity of the intestinal microbiota community was different between the CSJ and HC groups (Figure 5B).

### 3.4. Differences of microbiota genera in the CSJ and HC groups

**FIGURE 6:**
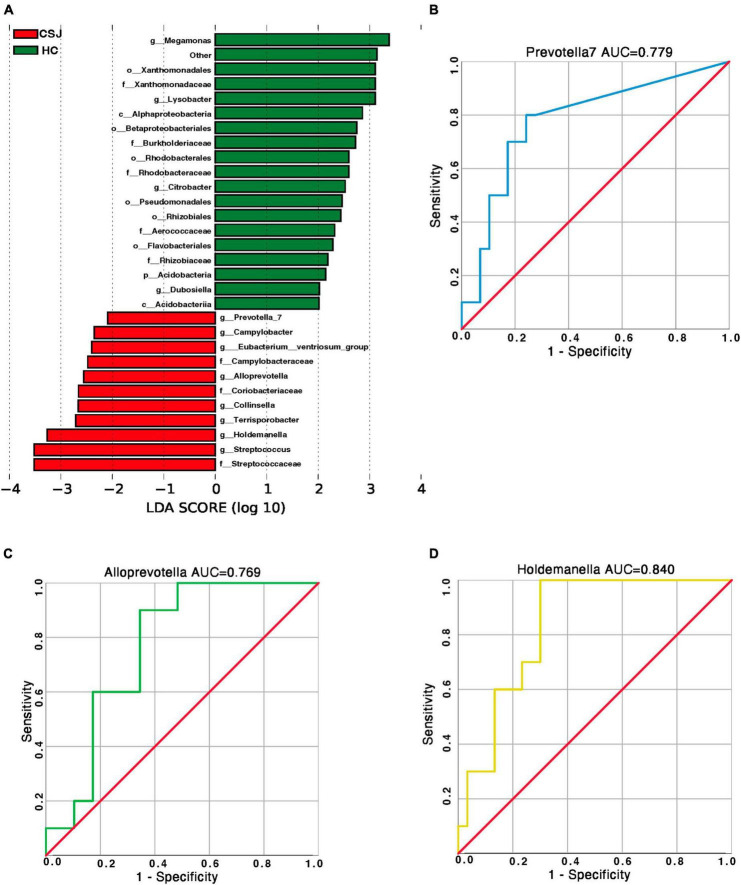
Species-specific differences in the intestinal microbiota between CSJ group and HC group. **(A)** The histogram of LDA scores showed the significantly different abundance of intestinal microbiota between the CSJ group and HC group. **(B–D)** The ROC analysis was done to predict the diagnostic efficiency of *Prevotella* 7, *Alloprevotella*, and *Holdemanella* genera for CSJ, and the AUC of *Prevotella* 7, *Alloprevotella*, and *Holdemanella* genera was 0.779, 0.769, and 0.840, respectively.

Linear discriminant analysis effect size was used to further explore the microbiota genera that differed significantly between the two groups. The abundance of more than 20 microbiota differed, however, some of these exhibited too low abundance at the family level to perform a clinical function. Combined with their relative abundance, *Prevotella* 7, *Alloprevotella*, and *Holdemanella* genera were significantly higher in the CSJ group than in the HC group (Figure 6A). We also did ROC analysis to explore the diagnostic efficiency of the *Prevotella* 7, *Alloprevotella*, and *Holdemanella* genera by SPSS. The results showed that the area under the curve (AUC) of *Prevotella* 7, *Alloprevotella*, and *Holdemanella* genera was 0.779, 0.769, and 0.840, respectively (Figures 6B–D), which indicated a potential value in non-invasive diagnosis of chronic schistosomiasis *japonica*-induced fibrosis.

### 3.5. The functional prediction of different microbiota genera

**FIGURE 7:**
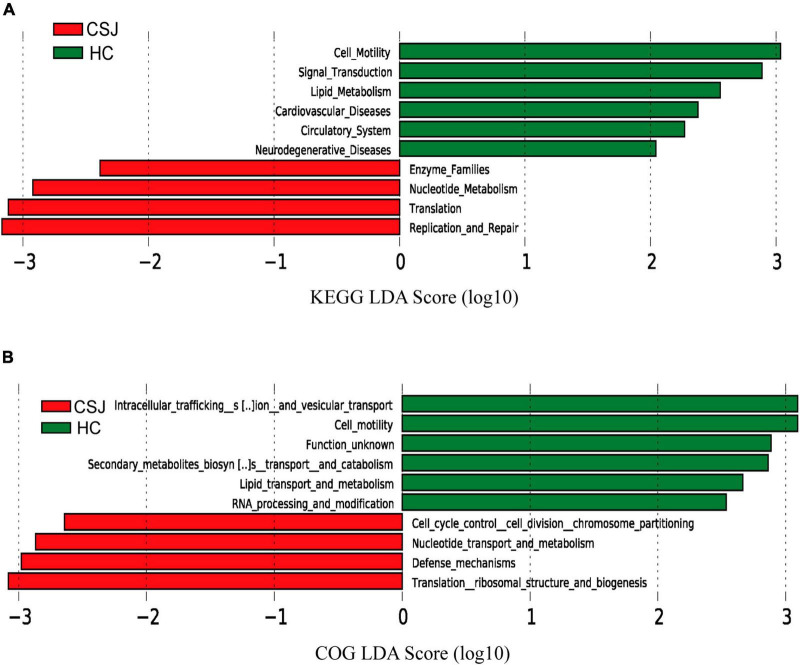
The functional prediction of different intestinal microbiota between CSJ group and HC group. The histogram of LDA scores showed the function of different intestinal microbiota between the two groups based on KEGG database **(A)** and COG database **(B)**.

Kyoto Encyclopedia of Genes and Genome and COG analyses were used to predict the function of the different intestinal microbiota in patients with chronic schistosomiasis *japonica*. KEGG analysis revealed that the Replication and Repair, and Translation pathways were significantly higher in the CSJ than in the HC groups (Figure 7A). COG analysis identified enrichment in the translation ribosomal structure and biogenesis and defense mechanisms after *S. japonicum* infection (Figure 7B).

## 4. Discussion

Schistosomiasis is a global disease that remains a significant threat to public health. *S. japonicum* is largely endemic in China, especially in the Dongting Lake District, negatively impacting human health and causing economic loss (Li et al., 2020; Wang et al., 2021). The current study analyzed the intestinal microbiota of the residents of Qingshan Island and is the first to identify the characteristics of intestinal microbiota among patients with chronic schistosomiasis *japonica*.

In our study, composition and abundance of intestinal microbiota at the phylum and family levels were different between two groups. The α and β diversity results showed that CSJ group had low diversity of species of the intestinal microbiome. *Prevotella* 7, *Alloprevotella*, and *Holdemanella* genera were markedly increased during the development of chronic schistosomiasis *japonica*. Moreover, the AUC of *Prevotella* 7, *Alloprevotella*, and *Holdemanella* genera was 0.779, 0.769, and 0.840, respectively, which indicated that the three intestinal genera had a potential value in diagnosis of chronic schistosomiasis *japonica*-induced fibrosis.

Both *Prevotella* 7 and *Alloprevotella* genera belong to the Prevotellaceae family, a Gram-negative obligate anaerobe that is a common component of intestinal microbiota. While these bacteria help digest protein and carbohydrate foods and are often defined as probiotics (Yeoh et al., 2022), they are also associated with chronic inflammatory conditions and intestinal diseases, such as ulcerative colitis (Ley, 2016). A recent study found that *Prevotellaceae* abundance was reduced in hepatitis B patients who developed cirrhosis (Li et al., 2022). Another study reported that Prevotellaceae family and *Prevotella* genus were significantly increased and considered as biomarkers in antituberculosis drugs-induced hepatotoxicity (Wu et al., 2022). Chen et al. (2022) verified that mosapride could reduce intestinal bacterial translocation by regulating the intestinal microbiota (main *Alloprevotella*) in rats with hepatic cirrhosis. *Holdemanella* genera may exhibit anti-inflammatory activity (Pujo et al., 2021). A recent study indicated that liver fibrosis was strongly correlated with the enrichment of *Prevotella* and *Holdemanella* genera (Kwan et al., 2022). Another study about severe acute kidney injury showed that amoxicillin treatment accelerated functional recovery and decreased kidney fibrosis after acute kidney injury by depleting *Holdemanella* genera (Gharaie et al., 2023). These findings suggest that the Prevotellaceae family and *Holdemanella* genera is closely associated with liver disease and may contribute to the development of chronic schistosomiasis *japonica*-induced fibrosis.

Kyoto Encyclopedia of Genes and Genome functional prediction analysis is an effective tool to study functional metabolic changes that help community samples to adapt to environmental changes, while COG is a common protein function classification database for prokaryotes that complements KEGG to reveal the functional composition of microbiota (Kanehisa and Goto, 2000; Galperin et al., 2019). Using these analyses, the Replication and Repair, and Defense Mechanism pathways were identified as the most prominent. *S. japonicum*-associated liver injury occurs as a result of repeated immune damage from the host granulomatous response to Schistosome eggs (Colley et al., 2014; Llanwarne and Helmby, 2021), which was consistent with the KEGG and COG results. These findings suggest that the intestinal microbiota plays an important role in liver damage repair.

The current study has some limitations. While Qingshan Island was chosen due to its high prevalence of *S. japonicum* infection, the collection of samples from this district alone may have introduced selection bias. In addition, the implementation of effective schistosomiasis control measures in China and low *S. japonicum*-associated morbidity rates limited the availability of large samples.

## 5. Conclusion

This study was the first to explore differences in the intestinal microbiota of patients with chronic schistosomiasis *japonica*-induced fibrosis and healthy people from Qingshan Island. *Prevotella* 7, *Alloprevotella*, and *Holdemanella* genera were significantly increased during the development of chronic schistosomiasis *japonica*-induced fibrosis, indicating a potential value in non-invasive diagnosis of chronic schistosomiasis *japonica*-induced fibrosis. Further studies using larger and more heterogeneous populations were needed to evaluate the value of clinical application.

## Data availability statement

The data presented in the study are deposited in the NCBI repository, https://www.ncbi.nlm.nih.gov/bioproject/PRJNA1018653, accession number PRJNA1018653.

## Ethics statement

The studies involving humans were approved by the Ethics Committee of The Third Xiangya Hospital of Central South University. The studies were conducted in accordance with the local legislation and institutional requirements. The participants provided their written informed consent to participate in this study. Written informed consent was obtained from the individual(s) for the publication of any potentially identifiable images or data included in this article.

## Author contributions

CG: Data curation, Formal analysis, Investigation, Methodology, Writing—original draft, Writing—review and editing. PZ: Funding acquisition, Writing—original draft, Writing—review and editing. JL: Data curation, Investigation, Writing—review and editing. CZ: Data curation, Investigation, Writing—review and editing. ZY: Data curation, Investigation, Writing—review and editing. YZ: Data curation, Investigation. YL: Data curation, Investigation, Writing—review and editing. JZ: Resources, Writing—review and editing. YC: Resources, Writing—review and editing. YM: Conceptualization, Funding acquisition, Project administration, Supervision, Writing—review and editing.
